# Entero-Enteric Fistula Following Multiple Magnet Ingestion in Children: A Systematic Review

**DOI:** 10.3390/jcm14176235

**Published:** 2025-09-03

**Authors:** Natalia Kelaidi, Konstantina Dimopoulou, Dimitra Dimopoulou, Aggeliki Krikri, Christina Siouli, Maria M. Berikopoulou, Nikolaos Zavras, Anastasia Dimopoulou

**Affiliations:** 1 Department of Pediatric Surgery, Children’s General Hospital “Aghia Sophia”, 115 27 Athens, Greece; kelaidinatalia@gmail.com (N.K.); akrikri@gmail.com (A.K.);; 2 Department of Gastroenterology, Korgialenio-Benakio Red Cross Hospital, 115 26 Athens, Greece; conu_med@hotmail.com; 3 Second Department of Pediatrics, Children’s General Hospital “Aglaia & Panagiotis Kyriakou”, School of Medicine, National and Kapodistrian University of Athens, 115 27 Athens, Greece; dimi_med@hotmail.com; 4 Second Department of Pediatrics, Children’s General Hospital “Aghia Sophia”, 115 27 Athens, Greece; maria-berikopoulou@hotmail.com; 5 Department of Pediatric Surgery, Attikon University Hospital, School of Medicine, National and Kapodistrian University of Athens, 115 27 Athens, Greece; nzavras@med.uoa.gr

**Keywords:** entero-enteric fistula, magnet ingestion, neodymium magnets, pediatric surgery, gastrointestinal complications, children

## Abstract

**Background/Objectives:** Entero-enteric fistula (EEF) formation following multiple magnet ingestion is a rare but severe complication among pediatric patients. The widespread availability of neodymium magnets in toys has increased incidence of ingestion and subsequent gastrointestinal injuries. This systematic review aims to summarize the clinical features, diagnostics, management and outcomes of pediatric EEF cases related to magnet ingestion and report our institution’s experience with four such cases. **Methods:** A systematic review was conducted using PubMed/Medline (January 1995–February 2025), focusing on EEF after ingestion of ≥2 magnets in patients ≤18 years old. Studies reporting original EEF cases were included. Data extraction included demographics, clinical presentation, diagnostic imaging, intervention type, fistula number/location, hospital stay, complications and outcomes. Four institutional cases were also analyzed. **Results:** Sixty-nine studies encompassing 130 pediatric patients were included. Median age was 3.3 years; 58% were male. The most common symptoms were abdominal pain (43%) and vomiting (29%). Abdominal X-ray identified magnets in all cases. Surgical intervention was required in 95.5%, while 5.5% were treated endoscopically. Ileal and jejunal fistulas were most common. Postoperative complications occurred in 19%, including bowel obstruction, infection and one death. Our four cases, aged 2 months to 5 years, each required surgery, with one patient readmitted for obstruction managed conservatively. **Conclusions**: Despite the case heterogeneity of this review, EEF is a potentially life-threatening complication of multiple magnet ingestion in children. Prompt diagnosis with abdominal X-ray and timely surgical management are essential. Increased clinical suspicion and public awareness are crucial for prevention and early intervention.

## 1. Introduction

Foreign body ingestion is a frequent problem among children especially between the age of 6 months and 3 years old, with mild predominance in males [1,2,3]. Multiple magnet ingestion is uncommon, but it might be dangerous due to possible serious events, resulting in bowel obstruction, perforation, fistula formation, ulceration and volvulus of the small and large intestine.

Magnet ingestion, in particular, represents a unique clinical challenge compared to other types of foreign bodies. High-powered neodymium magnets are several times stronger than conventional magnets, and when more than one is ingested or a single one combined with a metallic object, their powerful attraction across bowel loops can entrap intestinal walls (“sandwich” effect), leading to gastrointestinal damage by. More specifically, the pressure applied to the intestinal walls is enough to cause local ischemia, leading to localized necrosis, intestinal perforation or fistula formation, followed by peritonitis [4,5].

Entero-enteric fistula (EEF) formation, defined as abnormal communication between two parts of the bowel, is a relatively uncommon complication of multiple magnet ingestion among children. In this condition, an abnormal opening in the stomach or intestines allows the contents to leak into another section of the intestines [6]. Eventually, progressive bowel occlusion occurs, presenting with non-specific symptoms such as abdominal pain and vomiting, which can result in delayed diagnosis. In the existing literature, reports of EEF following multiple magnet ingestion are limited in the pediatric population [4,5].

Unlike coins or other inert objects that typically pass spontaneously, magnet ingestion often requires endoscopic or surgical intervention and is associated with significant morbidity and even mortality. Moreover, despite repeated regulatory actions and safety warnings, the incidence of magnet ingestion continues to rise, highlighting its importance as a pediatric public health concern that necessitates increased awareness among clinicians, caregivers, parents and policymakers. In order to reduce the incidence of occurrence of magnet ingestions, the Consumer Product Safety Commission (CPSC) of the United States forced the manufacturer of Buckyballs to recall them, by suing them in 2012. Additionally, the North American Society for Pediatric Gastroenterology, Hepatology, and Nutrition (NASPGHAN), proposed a ban for high powered neodymium magnets sale [6]. Nevertheless, in the Western world, the incidents of ingestions of high-powered neodymium magnets are rising annually [7,8,9].

This review summarizes the available literature on the clinical features, diagnosis and management of this unusual yet challenging condition in children, in order to raise clinical suspicion and awareness. Early diagnosis of EEF following multiple magnet ingestion is essential for effective treatment and improved outcomes. Additionally, we describe our related clinical experience in four cases of EEF.

## 2. Cases Presentation

A total of four children, one male and three females, were diagnosed with EEF following multiple magnet ingestion at the Department of Pediatric Surgery, Agia Sofia Children’s Hospital in Athens, Greece between January 2018 and February 2025. Their ages ranged from 2 months to 5 years old, while the number of magnets ingested was 23, 10, 5 and 5, respectively. All patients presented to the emergency department with abdominal pain and vomiting. The magnets were detected in all of our patients with abdominal X-rays. A CT scan and ultrasound were performed in one patient as part of the clinical evaluation. Initially, endoscopic intervention was performed in three children, but finally all four required laparotomies. Intraoperatively, two out of four children were diagnosed with single EEF, one with two EEFs and one with three EEFs, all located in different regions. Two EEFs were jejunocecal, three were jejunojenunal, one was jejunoileal and one jejunocolic (sigmoid colon). Two patients underwent intraoperative C-arm fluoroscopy. Although a remaining magnet was identified in one patient, no further surgical interventions were needed. The length of hospitalization ranged from 9 to 14 days, and one patient required readmission within the first month due to bowel obstruction that was treated conservatively.

## 3. Methods

### 3.1. Search Strategy

A systematic literature review was conducted by two independent reviewers using the electronic database of PubMed/Medline, Scopus and Web of Science focusing on entero-enteric fistula (EEF) formation following multiple magnet ingestion in children covering a period from January 1995 to February 2025. The keywords used were “magnet ingestion”, “fistula”, “children”, “pediatric”, “fistula” and “intestine”. The search terms were combined with the Boolean operators AND/OR. The keywords were used in all possible combinations to obtain the maximal number of articles. Furthermore, the reference lists of retrieved articles were manually reviewed to identify related studies. The literature search was restricted to the English language. All articles selected for inclusion were critically evaluated.

The PRISMA 2020 checklist is available in Tables S1 and S2 of the Supplementary Material.

### 3.2. Study Selection

In the first step of study selection, we ruled out irrelevant studies by assessing the titles and abstracts of the articles. Afterwards, we screened the full texts of the selected studies and included only those that met the following criteria: (1) Pediatric patients (≤18 years old), (2) Ingestion of two or more magnets leading to EEF formation. (3) Original research articles, case reports and case series, including both retrospective and prospective observational studies. (4) Studies reporting on the incidence, clinical presentation, diagnostic methods, management strategies and patient outcomes related to EEF formation. (5) Articles written in English.

Review articles, systematic reviews, meta-analyses, editorials, expert opinions and conference abstracts were not considered. Additionally, studies on foreign body ingestion unrelated to multiple magnets, articles that did not specifically describe EEF formation following multiple magnet ingestion or those that focused on other complications were excluded. After the first screening, a title/abstract and full-text screening was performed by two independent researchers for eligibility. Any disagreements between the two reviewers were resolved by a discussion reaching consensus.

### 3.3. Data Extraction

A standardized form was used to extract data from the included studies. Data were extracted independently by the two reviewers. The following information was extracted and recorded in the database: name of first author, year of publication, age, gender, number of ingested magnets, clinical symptoms, method of treatment, number and location of EEF, the use or not of intraoperative C-arm fluoroscopy, length of hospital stay and outcome. Categorical variables were summarized as absolute and relative (%) frequencies, while continuous variables as median and interquartile range, since they were skewed (normality was tested).

## 4. Results

**Figure 1:**
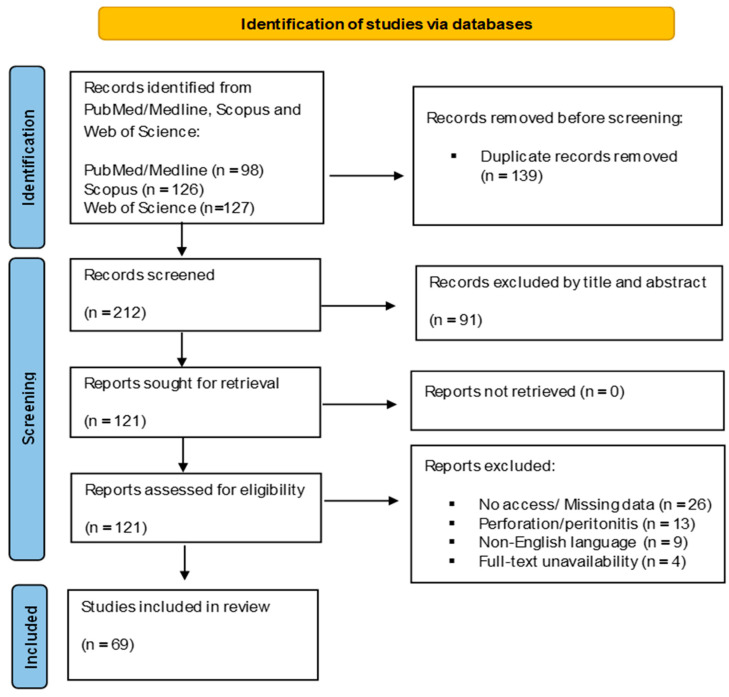
PRISMA 2020 flow chart illustrating the review process and article inclusion criteria.

A total of 98 articles were identified through the literature. After title and abstract evaluation, 24 articles were excluded, while 20 additional records were excluded after full-text review for not addressing entero-enteric fistula cases following multiple magnet ingestion (Figure 1). In total, the literature search revealed 69 published articles and 130 reported cases of pediatric patients with EEF [4,5,6,10,11,12,13,14,15,16,17,18,19,20,21,22,23,24,25,26,27,28,29,30,31,32,33,34,35,36,37,38,39,40,41,42,43,44,45,46,47,48,49,50,51,52,53,54,55,56,57,58,59,60,61,62,63,64,65,66,67,68,69,70,71,72,73,74,75] (Table 1).

The clinical and procedural characteristics of the patients enrolled in our study are presented in Table 2. The median age of our sample size was 3.3 years (ranging from 0.75 to 16 years). Moreover, the median number of ingested magnets was 11 (ranging from 1 to 62), while the vast majority of the children presented to Emergency Pediatric Department with abdominal pain and vomiting. Of note, only 11 patients were asymptomatic, with the magnets found accidentally in abdominal X-rays. In all cases, the ingested magnets were identified by abdominal X-rays (Table 2).

Regarding EEF treatment, no patient was treated conservatively. In all cases, EEF was recognized during endoscopic or surgical intervention. As a result, 6 patients (4.6%) were treated endoscopically, 104 (80%) underwent laparoscopy or laparotomy, while 20 (15%) were subjected to endoscopy followed by abdominal surgery (Table 2). Out of 124 patients who underwent laparoscopy or laparotomy, only 23 (18.5%) underwent intraoperative C-arm fluoroscopy to investigate possible remaining magnets in the abdominal cavity (Table 1).

**Figure 2:**
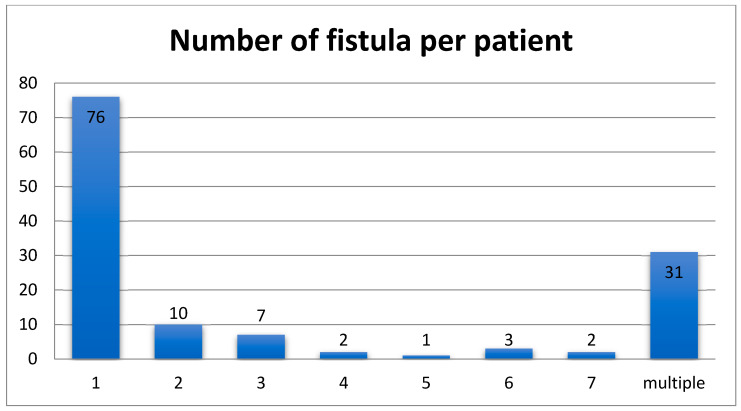
The number of fistulas per patient found in this systematic review.

A single EEF was identified in 76 patients, two EEFs in 10 patients, more than two EEFs in 15 patients; multiple EEFs were found in 31 patients (the specific number of them is not reported) (Figure 2). Moreover, regarding the EEF location, the most common type was ileoileal, presented in 32 patients (22%), followed by jejunoileal EEFs (27 patients, 19%). The various reported types of EEFs are illustrated in Table 3. Also, small-to-small bowel and small-to-large bowel EEFs were recognized in 13 and 3 children, respectively, without the authors reporting their exact location. Finally, in 46 cases (35%) the location of the fistula was not mentioned (Table 3).

The median duration of hospitalization was eight days (ranging from 1 to 42) for patients that were treated surgically compared to two days for those treated solely endoscopically. Only nine patients (7%) required a prolonged hospital stay. Regarding the patients’ outcome, the majority of patients (72%) had an uneventful post-operative course and follow-up period, while 22 children (17%) experienced complications. Due to these postoperative events, nine patients underwent repeat endoscopy and six re-operation (Table 4).

## 5. Discussion

The proportion of high-powered neodymium magnets ingestion by children is continuously increasing due to their wide availability as parts of toys, tools or entertainment products [9,76]. It is important to point out that high powered neodymium magnets compared to simple iron magnets are 5 to 20 times more powerful, thus the complications after their ingestion might be severe and fatal. Approximately 80–90% of ingested FB (except for neodymium magnets) goes right through the whole gastrointestinal tract without any event. A proportion of 10 to 20% might need endoscopic removal and less than 1% need surgical intervention [77]. EEF formation is a severe complication of multiple magnets ingestion in children and can be fatal in some cases. This systematic review of the literature sheds light on this uncommon manifestation, evaluates the efficacy of diagnostic and treatment methods and assesses the outcomes.

Our results and clinical experience revealed that the overwhelming majority of patients with EEF following multiple magnet ingestion present with vomiting or abdominal pain. Our review indicates that patients rarely present with severe symptoms such as signs of peritonitis, fever, anuria or bowel obstruction, whereas seven children were asymptomatic. These findings could be potentially attributed to parents or guardians observing the magnet ingestion, the early onset of symptoms, the immediate seeking for medical advice and the prompt diagnosis and effective treatment. On the other hand, the presentation with mild non-specific symptoms, the lack of witness to the magnet ingestion or the misdiagnosis, as the magnets are tightly attached and might appear as a single object, could lead to delayed diagnosis and treatment increasing the risk of life-threatening complications [4,9].

Plain abdominal X-ray remains the gold standard for diagnosing magnet ingestion in children and should always be performed when foreign body ingestion is reported or suspected [38]. In all cases, the ingested magnets were detected by abdominal X-ray. Moreover, there are some limitations of this diagnostic tool, especially in cases of multiple magnet ingestion, as multiple magnets could be bound together and perceived as a single object, like in one of our cases. Therefore, multiple radiologic views are recommended (Face and Profile) [33]. North American Society for Pediatric Gastroenterology, Hepatology, and Nutrition (NASPGHAN) members, recognized the severity of this issue and its significant morbidity, and developed an algorithm in 2015 contributing to the accurate diagnosis of children with magnet ingestion. According to these recommendations, multiple magnets detected in the stomach should be removed endoscopically, while those located beyond the stomach should be managed based on symptoms and magnet progression. In asymptomatic patients with no obstruction or perforation on X-ray, the progression of the magnets may be followed with serial X-rays. The presence of symptoms and lack of magnet progression are absolute indications for surgical intervention [78]. Our review highlights that X-ray is the only diagnostic tool used in the management of patients with multiple magnet ingestion. Sodagum et al. proposed the “signet ring” appearance on abdominal X-ray, as a preoperative diagnostic sign of EEF in cases of magnet ingestion. They suggested that its presence alone should be an indication for surgical intervention [61]. Based on our clinical experience, all four patients underwent surgery following abdominal X-ray, due to severe symptoms and the number of ingested magnets. EEF was diagnosed intraoperatively, as neither CT scan nor ultrasound had raised suspicion of its presence.

Our results demonstrate the effectiveness of the endoscopic method in the management of children with EEF as all patients, who were subjected to endoscopic intervention, were successfully treated with no complications and short duration of hospitalization. More specifically, in five out of these six children, the fistula was left untreated and patients had a close follow up, while one was treated with the placement of an endoclip. Of note, 10 out of 124 patients who underwent surgery were treated laparoscopically by suturing the fistulas and removing the magnets by enterotomies. None of them had any complications and their hospital stay was on average five days. The rest of the patients underwent exploratory laparotomy due to intestinal torsion, multiple fistulae, multiple perforations or abscesses, and were treated by enterotomies and suturing or enterectomies and anastomosis. Finally, the complexity of the treatment of this condition is illustrated by the significant percentage of children (17%) who developed complications, such as SSI, abdominal wall dehiscence, bowel obstruction, anastomotic leak, pneumonia and death.

For the possibility of remaining magnets, a C-arm fluoroscopy intraoperatively is indicated [78]; however, despite the current recommendations, only 23 patients underwent intraoperative fluoroscopy. C-arm fluoroscopy is an important diagnostic and must be always applied in order to prevent further investigation and re-operation.

Our study has several strengths. This systematic review includes a broad range of studies on EEF formation following multiple magnet ingestion in children. It covers a long time period incorporating the most up-to-date research on this uncommon and serious entity. Additionally, the review includes a total of 130 reported cases from 69 articles. This large sample size increases the reliability of the results providing essential information about clinical presentation, treatment strategies and outcomes associated with multiple magnet ingestion and EEF formation. In addition to the literature review, the study involves our clinical experience with four pediatric patients diagnosed with EEF following multiple magnet ingestion indicating how the pediatric patients can be treated in a real-world setting. Finally, our study describes both the short-term and long-term outcomes of patients, highlighting complications and risks associated with treatment and recovery.

However, our study has some limitations. First, there is significant heterogeneity in the number of patients, the type and number of magnets ingested reported between the studies, which could result in variability in the findings. Second, in 35% of the cases, fistula location was not reported, limiting our knowledge of the typical anatomical sites of EEFs following magnet ingestion that could effectively guide surgical decisions. Third, most of the studies included in the review are case reports or case series that are prone to publication bias. Due to the rarity and urgency of the condition, it is challenging to design randomized controlled trials and high-quality study designs that would enhance the quality of available evidence.

Finally, according to our findings and institutional experience, we suggest a pragmatic approach to the management of multiple magnet ingestion complicated by EEF. An abdominal X-ray should be performed in all suspected cases, as it reliably identifies ingested magnets. Computed tomography should be reserved for patients with unclear findings and suspicion of perforation but should not delay intervention. Endoscopic retrieval should be attempted when magnets are located in the esophagus or stomach in absence of perforation or obstruction. Once magnets have passed beyond the stomach, endoscopic retrieval is rarely successful, and close monitoring with serial X-rays or early surgical exploration is warranted. In symptomatic patients or when improvement is not observed, surgical intervention should be performed. Laparoscopy may be considered in stable children with limited findings, while laparotomy remains the procedure of choice in patients with multiple fistulae, perforation or peritonitis.

## 6. Conclusions

During the last two decades, an increase in the report of cases of children with incidental ingestion of multiple magnets has been observed. The diagnosis may be difficult and delayed, because of the late onset of the symptoms, usually non-specific ones and the unwitnessed cases in majority. Special awareness should be given by medical practitioners, as the complications after multiple magnet ingestion or co-ingestion of a single magnet and a metallic object could be serious. Among these major complications is the formation of EEF, which requires prompt and appropriate surgical treatment. Finally, the establishment of new regulations and safety standards to prevent these kinds of accidents is necessary.

This systematic review represents the largest synthesis of pediatric EEF cases following multiple magnet ingestion to date, encompassing 130 reported cases and four additional institutional cases. The pooled data highlights consistent diagnostic and therapeutic patterns, with abdominal X-ray identifying magnets in all patients and timely surgical or endoscopic intervention leading to favorable outcomes in the majority. These findings not only consolidate current knowledge but also provide practical guidance for clinicians, underscoring the critical importance of early recognition and intervention to reduce morbidity and mortality.

## Figures and Tables

**Table 1:** Baseline characteristics of the patients included in the systematic review.

Study (Year) [Reference]	No. of Patients with Fistula	No. of Fistula	Gender (M/F)	Age (y) ^a^	Intervention	Hospital Stay ^a^	C-Arm Fluoroscopy
Mostafa et al. (2021) [10]	8	23 (3 ^a^)	N/A	5.8	1E + LP, 6LP, 1LS + LP	10	2
Chavan et al. (2022) [11]	3	3	1/2	2	E	1	-
Seguier-Lipszyc et al. (2022) [12]	1	1	1/-	6	LS + LP	7	-
Blevrakis et al. (2018) [6]	1	1	1/-	9	LP	14	-
Mandhan et al. (2014) [13]	2	2 (1 ^a^)	1/1	3	2LS + LP	14	1
Muniz Dias et al. (2019) [14]	1	1	1/-	10	LP	4	-
Merchant et al. (2017) [15]	1	1	1/-	2	E	4	1
Robinson et al. (2009) [16]	1	1	1/-	3	LP	N/A	-
Khan et al. (2023) [17]	4	6 (2 ^a^)	1/3	2.1	1E + LP, 1LS + LP,2LP	N/A	-
Shah et al. (2009) [18]	16	21 (1 ^a^)	10/6	3.9	1E, 1E + LP,14LP	12	2
Pryor et al. (2007) [19]	1	2	1/-	12	LP	-	1
Honzumi et al. (1995) [20]	1	1	-/1	3	LP	-	-
Kubota et al. (1995) [21]	1	1	-/1	1.5	LP	7	-
Lee et al. (1996) [22]	2	2 (1 ^a^)	-/2	2.5	LP	-	-
Chung et al. (2003) [23]	2	2 (1 ^a^)	2/-	1.5	LP	8	-
Haraguchi et al. (2004) [24]	1	1	1/-	2	LP	8	-
Nui et al. (2005) [25]	1	1	1/-	1	LP	21	-
Liu et al. (2005) [26]	1	1	1/-	7	E + LP	-	-
Ohno et al. (2005) [27]	1	1	-/1	1	E	-	-
Vijaysadan et al. (2006) [28]	1	2	1/-	11	LP	-	-
Uchida et al. (2006) [29]	1	3	-/1	2	LP	28	-
Anselmi et al. (2007) [30]	1	1	1/-	3	LP	-	-
Oestreich et al. (2006) [31]	1	2	1/-	4	LP	2	-
Adikibi et al. (2013) [32]	1	2	-/1	3	LS + LP	6	-
Waters et al. (2015) [33]	37	N/A	25/12	N/A	N/A	N/A	N/A
Pederiva et al. (2014) [34]	1	1	1/-	4	LS + LP	5	-
Dutta et al. (2008) [35]	1	1	1/-	4	LS	3	-
Zachos et al. (2019) [36]	1	2	1/-	4	LP	5	-
Taher et al. (2019) [37]	1	7	1/-	4	LP	6	-
Cherchi et al. (2018) [38]	1	1	N/A	11	LP	8	1
Wooten et al. (2012) [39]	1	1	1/-	16	LS + LP	8	-
Wang et al. (2020) [40]	2	4 (2 ^a^)	N/A	3	N/A	N/A	N/A
Miyamoto et al. (2019) [5]	1	1	1/-	3	E + LP	6	-
Tsai et al. (2013) [41]	1	1	-/1	1	E + LS + LP	N/A	-
Saeed et al. (2009) [42]	1	1	1/-	11	LP	N/A	-
Kosut et al. (2013) [43]	3	4 (1 ^a^)	3/-	3.3	3E + LS	4	3
Pogorelić et al. (2016) [44]	1	1	-/1	2	LP	5	-
Ali et al. (2020) [45]	1	1	-/1	9	E + LP	5	1
Martinez et al. (2021) [46]	2	10 (5 ^a^)	2/-	6.5	2LLP	7	-
Gun et al. (2013) [47]	1	1	-/1	2	LP	7	-
Zheng et al. (2021) [48]	1	1	N/A	N/A	N/A	N/A	-
Phen et al. (2018) [49]	1	1	1/-	1.5	E	3	-
Ahmed et al. (2010) [50]	1	1	-/1	5	E + LP	6	-
Kisku et al. (2015) [51]	1	1	1/-	2	LP	8	-
Kromhout et al. (2010) [52]	1	3	1/-	3	LP	4	-
Hesketh et al. (2014) [53]	1	7	1/-	10	LP	14	-
Clarke et al. (2010) [54]	1	MULTIPLE	1/-	8	LP	N/A	-
Cozzarelli et al. (2017) [55]	1	1	1/-	5	LP	7	-
CDC (2006) [56]	1	1	1/-	4	N/A	N/A	-
Chang et al. (2022) [57]	1	1	N/A	N/A	N/A	N/A	-
Kumar et al. (2024) [58]	2	MULTIPLE + 1	0/2	4	2LP	3	-
Quezada et al. (2023) [59]	1	1	0/1	1.5	E + LS + LP	5	1
Özcan et al. (2024) [60]	1	1	0/1	6	E + LP	8	-
Sodagum et al. (2024) [61]	2	4	2/-	4	E + LP, LP	8	1
Khan et al. (2024) [62]	1	1	N/A	6	LP	N/A	-
Alareefy et al. (2023) [63]	1	1	1/-	3	LP	3	-
George A.T et al. (2012) [64]	2	MULTIPLE + 1	N/A	5	2LP	N/A	-
Santos et al. (2019) [65]	1	1	1/-	1.5	LP	6	1
Nguyen et al. (2023) [66]	1	4	1/-	2	E + LS + LP	N/A	1
Kim et al. (2014) [67]	1	1	-/1	0.75	E + LP	N/A	-
Morulana et al. (2021) [68]	1	1	-/1	5	LS + LP	5	1
Azzam et al. (2023) [69]	1	8	1/-	5	LP	N/A	1
Lawrence et al. (2019) [70]	1	6	-/1	3	E + LP	N/A	1
Teague et al. (2013) [71]	1	1	1/-	1.5	E + LP	6	-
Mervin et al. (2020) [72]	1	2	-/1	4	LP	7	1
Elsherbeny et al. (2018) [73]	1	1	-/1	3	LP	>42	N/A
Afzal et al. (2022) [74]	3	5 (2 ^a^)	3/-	3	3LS	8	2
Si et al. (2016) [75]	1	1	1/-	2	LP	11	-
Al-Saied et al. (2022) [4]	2	MULTIPLE + 2	2/-	4.5	LP, LS + LP	6	2
Total	130	158	76/38	3.3			23

^a^ mean (SD). M: male; F: female; LP: laparotomy; LS: laparoscopy; E: endoscopy; N/A not available.

**Table 2:** Clinical characteristics of multiple magnet ingestion in children presenting with entero-enteric fistula.

Characteristics	Cases	Percentage (%)
Gender		
Female	38	29
Male	76	58
Clinical symptoms		
Abdominal pain	60	46
Vomiting	43	33
Abdominal obstruction	10	8
Diarrhea	4	3
Constipation	6	5
Acute abdomen	4	3
Anuric	2	2
Fever	4	3
Melena	1	1
None	11	8
Clinical interventions		
Endoscopic interventions	6	4.6
Endoscopic interventions and abdominal surgery	20	15
Abdominal surgery	104	80

**Table 3:** Fistula location in children after multiple magnet ingestion.

**Location ^1,2^**	**Number of Fistulas**	**Percentage (%)**
Gastric fistulas	24	17
Gastro-Esophageal	1	1
Gastro-Gastric	2	1
Gastro-Duodenum	3	2
Gastro-Jejunum	15	10
Gastro-Cecum	1	1
Gastro –Transverse colon	1	1
Gastro-Small bowel	2	1
Duodenal fistulas	10	7
Duodenum-Jejunum	8	6
Duodenum-Ascending colon	1	1
Duodenum-Transverse colon	1	1
Enteric fistulas	110	53
Jejunum-Jejunum	13	9
Jejunum-Ileum	27	19
Jejunum-Cecum	1	1
Jejunum-Transverse colon	3	2
Jejunum-Descending colon	1	1
Jejunum-Sigmoid colon	1	1
Jejunum-Colon	1	1
Ileum-Ileum	32	22
Ileum-Cecum	7	5
Ileum-Appendix	1	1
Ileum-Ascending colon	1	1
Ileum-Sigmoid colon	1	1
Ileum-Colon	5	3
Small Bowel-Small Bowel	13	9
Small Bowel-Large Bowel	3	2

^1^ Patients with multiple^1^ M^1^ Multiple fistulas are not included. ^2^ In 15 patients the location of fistula is not described.

**Table 4:** Postoperative period of children with entero-enteric fistula after multiple magnet ingestion.

**Postoperative Period**	**Cases**	**Percentage (%)**
Uneventful	94	72
Postoperative events ^1^	22	17
SSI	4	
Abdominal wall dehiscence	1	
Bowel obstruction	2	
Gastroesophageal junction stricture	1	
Anastomotic leak	5	
Pneumonia	1	
New endoscopy	9	
New surgery	6	
Death	1	
N/A	14	11

^1^ Seven patients showed with more than one postoperative event; N/A not available.

## Data Availability

The data underlying this article will be shared at reasonable request to the corresponding author.

## Supplementary Materials

The following supporting information can be downloaded at: https://www.mdpi.com/article/10.3390/jcm14176235/s1, Table S1: Preferred reporting items for systematic reviews and meta-analyses (PRISMA) 2020 abstract checklist; Table S2: Preferred reporting items for systematic reviews and meta-analyses (PRISMA) 2020 checklist.

## Abbreviations

EEF	Entero-enteric fistula
NASPGHAN	North American Society for Pediatric Gastroenterology, Hepatology, and Nutrition
